# Overall haemostatic potential (OHP) assay can risk stratify for venous thromboembolism recurrence in anticoagulated patients

**DOI:** 10.1007/s11239-022-02686-6

**Published:** 2022-07-31

**Authors:** Julie Wang, Hui Yin Lim, Rowena Brook, Jeffrey Lai, Harshal Nandurkar, Prahlad Ho

**Affiliations:** 1 Northern Health, 185 Cooper St Epping, Melbourne, VIC 3076 Australia; 2 Australian Centre for Blood Diseases, Melbourne, VIC 3004 Australia; 3 University of Melbourne, Parkville, VIC 3010 Australia

**Keywords:** Overall Haemostatic Assay, OHP, VTE recurrence risk stratification

## Abstract

Assessing the risk of recurrent venous thromboembolism (VTE), particularly when patients are anticoagulated, remains a major challenge largely due to the lack of biomarkers. Blood was sampled from adult VTE patients recruited between January 2018 and September 2020, while receiving therapeutic anticoagulation. Results were compared to 144 healthy subjects (34.7% male, median age 42 years). Overall haemostatic potential (OHP) assay, a spectrophotometric assay, was performed on platelet-poor plasma, in which fibrin formation (triggered by small amounts of thrombin (overall coagulation potential, OCP)) and fibrinolysis (by the addition of thrombin and tissue plasminogen activator (OHP)) are simultaneously measured. Results were obtained from 196 patients (52.6% male, mean age 57.1 years). Compared to healthy subjects, VTE patients displayed significantly higher OCP (39.6 vs 34.5 units, p < 0.001) and OHP (9.3 vs 6.4 units, p < 0.001) as well as lower overall fibrinolytic potential (75.6 v s81.1%, p < 0.001). All 16 VTE recurrences, including 11 unprovoked, occurred above an OCP cut-off of 40^th^ percentile (recurrence rate 4.32/100 patient-years (100PY), 95% confidence interval (CI) 2.39–7.80, p = 0.002). Of 97 patients who subsequently discontinued anticoagulation, all unprovoked VTE recurrences (n = 9) occurred above the 40^th^ OCP percentile (recurrence rate 9.10/100PY, 95% CI 4.74–17.49, p = 0.005) and the 40^th^ OHP percentile (recurrence rate 8.46/100PY, 95% CI 4.40–16.25, p = 0.009). Our pilot study demonstrates that the OHP assay can detect a hypercoagulable and hypofibrinolytic state in anticoagulated VTE patients and may be able to risk stratify VTE recurrence, allowing for more individualised decision on long-term anticoagulation. Further larger prospective studies are required.

## Highlights

- Compared to healthy subjects, VTE patients displayed significantly high OCP, OHP and lower overall fibrinolytic potential
- All 16 VTE recurrences occurred above the 40th OCP percentile
- Of patients who discontinued anticoagulation, all 9 unprovoked VTE recurrenced occurred above the 40th OCP and OHP percentiles
- OCP performed better than D-dimer in ability to predict unprovoked recurrent VTE (ROC AUC 0.71 vs 0.43)

## Introduction

Assessing the individual risk of recurrent venous thromboembolic (VTE) events remains one of the major challenges of VTE management. While large studies have reported up to 30% recurrence rate at 5 years following a first unprovoked proximal deep vein thrombosis (DVT) or pulmonary embolus (PE) [1], up to 70% of patients may not subsequently develop VTE recurrence [2]. Recommending indefinite anticoagulation to minimise the risk of recurrence is not without its risks and in situations of clinical equipoise, there is a need for an individualised and targeted approach to risk assessment.

Several biomarkers and assays have been explored as adjuncts to clinical decision making, and among the most well-known of these include D-dimer and thrombin generation assays [3], which have been associated with increased risk of VTE recurrence [4–9]. An important drawback of these biomarkers is the requirement to test when the patient has discontinued anticoagulation, which can be difficult in the VTE population as the risk of recurrent events is highest within the first 12 months following anticoagulation cessation [2, 10]. The only model to incorporate biomarker testing whilst anticoagulated is the HERDOO2 [11]. However, this score has been shown to lack discriminatory ability in males, with all males recommended for long-term anticoagulation following a first unprovoked VTE irrespective of their individual characteristics.

There has been a renewal of interest into examining the fibrinolytic system and its role in recurrence of VTE, with several recent studies demonstrating markers of reduced fibrinolysis including a prolonged clot lysis time (CLT) and low permeability coefficient (K_s_) to be independently associated with VTE recurrence following a first DVT [12] or PE [13]. The Overall Haemostatic Potential (OHP) assay is a global coagulation assay that can examine both fibrin production and lysis within the same test system [14]. This assay has been shown to be able to detect hypercoagulable states following PE [15] and pregnancy-associated VTE [16], as well as potentially predict thrombosis in other hypercoagulable states such as diabetes mellitus [17], ischaemic heart disease [18] and antiphospholipid syndrome [19]. However, its role in risk stratification of future VTE recurrence has not been previously explored.

We aimed to investigate the use of the OHP assay in newly diagnosed patients following VTE and explore its role in the risk stratification of VTE recurrence, including in patients whilst receiving anticoagulation.

## Methods

This is a prospective observational study of adult patients aged 18 years and over with VTE, recruited through the haematology outpatient clinics of a sub-tertiary hospital, Northern Hospital, Victoria, Australia. At the time of recruitment, patients were required to be in their active phase of VTE treatment with therapeutic anticoagulation (warfarin, rivaroxaban or apixaban). Exclusion criteria included superficial thrombophlebitis, unconfirmed VTE diagnosis without objective radiological evidence of VTE, significant anaemia and inability to undergo venepunctures at our institution. For this analysis, non-lower limb DVT and non-PE patients such as those with upper limb DVT, cerebral venous sinus thrombosis (CVST) and splanchnic vein thrombosis were also excluded. Baseline information for all patients were collected at the initial clinical visit including age, gender, characteristics of the VTE, risk factors for VTE, smoking status, weight, and their relevant co-morbidities. Written informed consent was obtained from every study participant. This study was approved by the Human Research Ethics Committee of Austin Health (Austin429) and Northern Health (HREC14).

All blood samples were obtained by trained phlebotomists using standard venepuncture techniques via a 21G needle while the participant was still receiving therapeutic anticoagulation. The duration of anticoagulation was determined by the clinician, in discussion with the patient. Routine investigations including full blood count, coagulation studies, D-dimer and renal function tests, thrombophilia screen (including protein C, protein S, antithrombin, factor V Leiden mutation, prothrombin gene mutation), lupus anticoagulant, anticardiolipin IgG and b2-glycoprotein-1 IgG were performed using standard accredited laboratory protocol. The STA® fibrinogen kit was used to measure the fibrinogen levels by the Clauss method. D-dimer was measured using the immunoturbidimetric method with the STA-LIATEST D-Di Plus kit. The remaining citrate samples were double centrifuged at 2500 g for 10 min to obtain platelet-poor plasma and the supernatant stored at − 80 °C within two hours of collection. These samples were then thawed at 37 °C and batch-tested for the OHP assay.

### Healthy subjects

The OHP results of the VTE cohort were compared to those of a previously published cohort of 144 healthy subjects (34.7% male, median age 42 years) [17, 20], using the same methodology as that described above. The inclusion criteria for healthy controls were stringent including no known cardiovascular risk factors and thrombosis history, medications such as anticoagulants, anti-platelets and oral contraceptives, as well as a negative thrombophilia screen.

### Overall haemostatic potential (OHP) assay

**Fig. 1:**
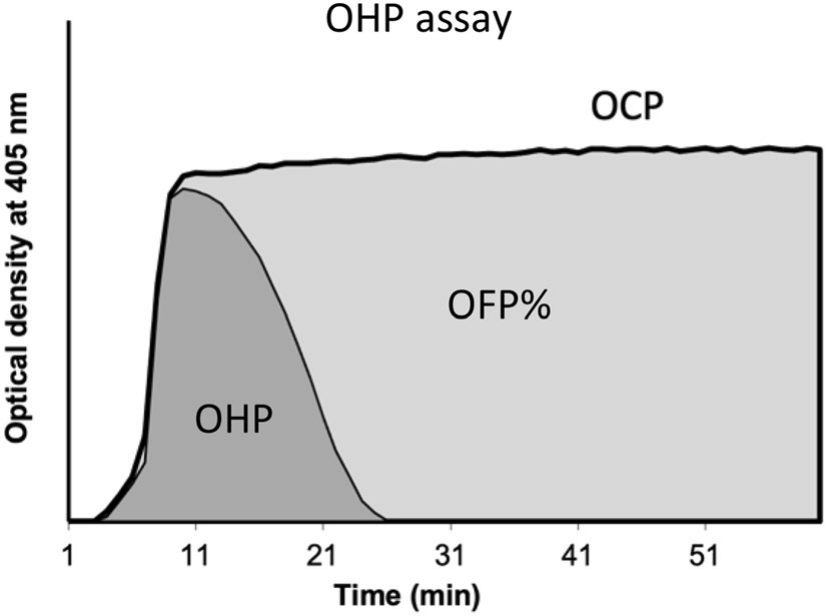
OHP assay. OCP is the area under the curve (AUC) of the OD curve obtained by the addition of thrombin (0.006 IU/ml) with CaCl2. OHP is the AUC of the curve by adding thrombin (0.006 IU/ml), CaCl2 and tPA. The OFP% is the difference between the AUCs OCP and OHP. OCP overall coagulation potential; OHP overall haemostatic potential; OFP% overall fibrinolytic potential

The OHP assay is derived from a fibrin aggregation curve formed from repeated spectrophotometric measurements of platelet-poor plasma (Fig. 1). 75 μL of thawed PPP was added to wells with 75 μL of buffer containing either (i) Tris, NaCl, CaCl2 (final concentration 66 nM Tris, 130 mM NaCl, 35 mL CaCl_2_; pH 7.0) and thrombin (0.006 IU/mL) to generate the overall coagulation potential (OCP) or (ii) Tris, NaCl, CaCl_2_, thrombin and tissue plasminogen activator (tPA) (600 ng/mL) to generate the OHP. The two fibrin-aggregation curves (OCP and OHP) are cumulatively calculated from the FLUOstar Optima (BMG Labtech) plate reader at 405 nM. The difference between the area underneath the two curves gives the overall fibrinolytic potential (OFP%).

Patients were followed-up every 3 months during the initial anticoagulation period, then 6–12 months for up to 3 years following anticoagulation cessation, or for 3 years from VTE diagnosis for those who remained on indefinite anticoagulation. Each follow-up visit involved review for recurrent VTE. Recurrent VTE was defined as a new episode or progression of DVT or PE, objectively measured by compression ultrasonography and/or computed tomography pulmonary angiography (CTPA) or ventilation/perfusion (V/Q) scan. Superficial thrombophlebitis was not classified as recurrent VTE. Patients were classified as falling into a ‘clinical equipoise’ group with respects to VTE recurrence risk if they were following a first unprovoked or minimally provoked major VTE (defined as proximal DVT and/or PE). Minimally provoking factors included minor transient factors including long-haul flights more than 4 h duration, lower limb injury with partial immobilisation or hospital admission for medical reasons.

### Statistical analysis

Statistical analysis was performed using Stata version 17.0 (StataCorp, College Stations, Texas, USA). Comparisons between patient groups were conducted using Student’s t-tests for those variables which were normally distributed and presented as means and standard deviation. Mann–Whitney (rank-sum) was performed for those variables found to be non-normally distributed and were presented as medians and interquartile ranges (IQR). Categorical variables were presented as counts and frequencies with χ^2^ tests to test for differences. For multivariate analysis, skewed variables were transformed into a normal distribution before linear regression was performed to account for confounders including age and sex. Receiver Operative Curve (ROC) analysis was used to determine the area-under-the-curve (AUC) for D-dimer and OCP. A two-tailed p-value of less than 0.05 was attributed statistical significance.

Time-to-event analysis was performed with recurrent VTE or recurrent unprovoked VTE as endpoints. Patients without VTE recurrence who were alive or lost to follow-up were censored at the date of last follow-up. Incidences of recurrence were compared between groups using cox proportional hazards regression analysis. Average rates of VTE recurrence are reported as events per 100 patient-years (100PY) to account for the differing lengths of follow up across patients. No deaths occurred in the subgroup without long-term anticoagulation, so this was not included as a competing risk.

## Results

**Fig. 2:**
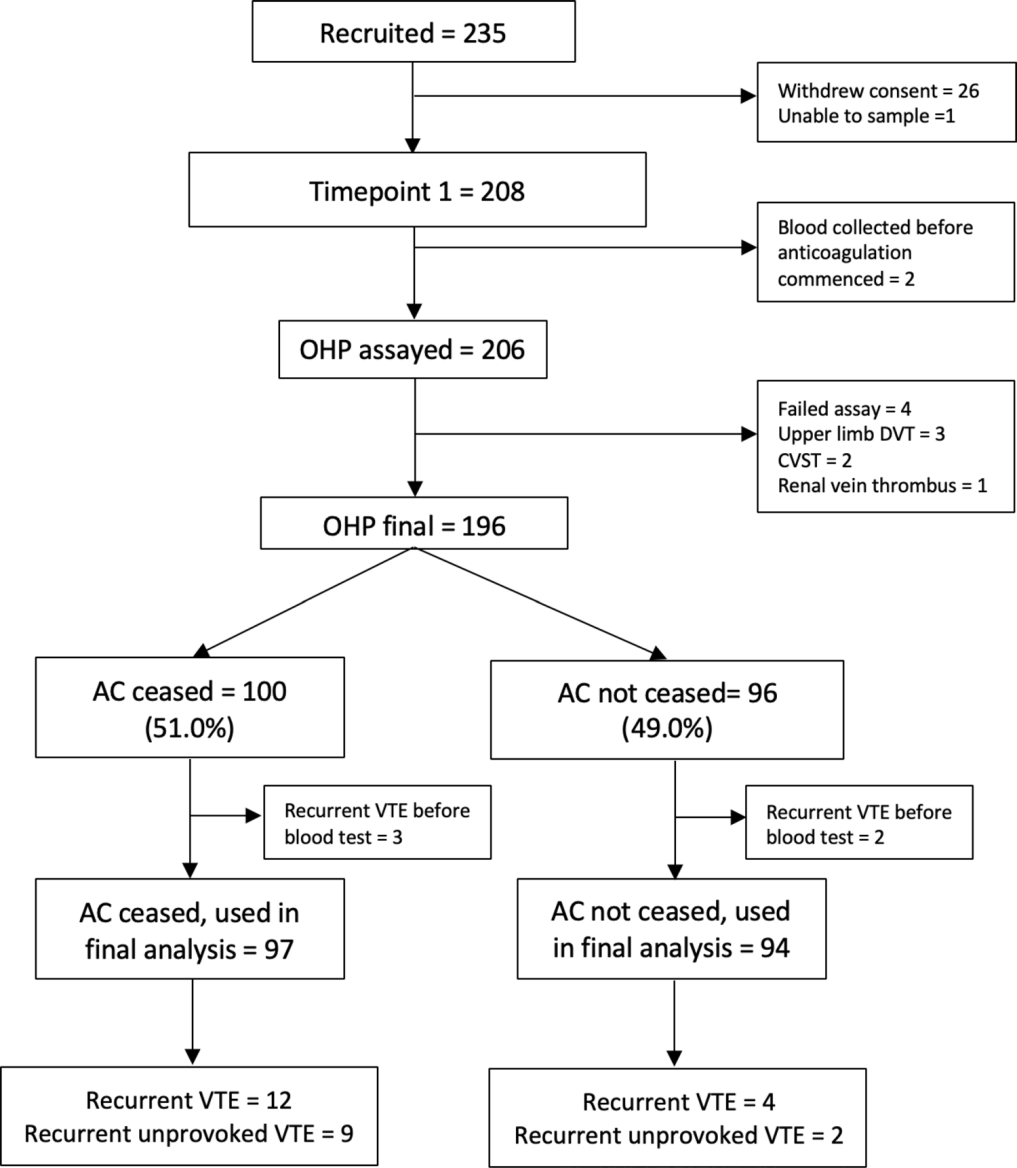
Consort diagram of study population. *AC* anticoagulation; *CVST* cerebral venous sinus thrombosis

Between January 2018 and September 2020, a total of 235 patients were recruited, of which 208 had blood sampled by the time of this interim analysis (Fig. 2). 196 samples analysed were included in the final analysis, after exclusion of 3 patients with upper limb DVT, 2 with CVST, 1 with renal vein thrombus, 4 with failed assays and 2 who had blood collected prior to anticoagulation commencement. Five patients who developed VTE recurrence prior to blood sampling were excluded from the time-to-event analysis, due to a significantly shorter time interval to blood sampling in this group, compared to patients who developed VTE recurrence following blood sampling (mean number of days 20.2 v 91.9, p = 0.013).

Baseline characteristics of patients are displayed in Table 1. The median time from VTE diagnosis to time of blood collection was 79 days (range 41–114 days). There were 92 (46.9%) patients with PE with or without DVT, 47 (24%) with proximal DVT alone and 57 (29.1%) with isolated distal DVT (IDDVT). Anticoagulation was subsequently discontinued in 100 (51.0%) patients. There were 16 VTE recurrences following blood collection. The median duration from time of diagnosis to VTE recurrence was 1.35 years (range 0.36–4.38 years).

**Table 1:** Baseline characteristics of patients who have continued or discontinued anticoagulation, and with and without VTE recurrence

	All	Discontinued anticoagulation	Indefinite anticoagulation	p-value*	No recurrence	VTE recurrence	p-value^#^
N	196	100	96		175	16^	
Age, mean (SD)	57.1 (14.3)	54.1 (13.9)	60.3 (14.1)	**0.003**	57.3 (14.4)	54.9 (12.9)	0.51
Male (%)	103 (52.6%)	49 (49.0%)	54 (56.2%)	0.31	95 (54.3%)	6 (37.5%)	0.20
Weight (kg), median (IQR)	92.0 (79.0, 105.0)	88.0 (76.0, 107.0)	93.0 (80.0, 104.0)	0.56	92.0 (78.5, 105.0)	97.5 (80.0, 118.5)	0.29
VTE type	139 (70.9%)	57 (57.0%)	82 (85.4%)	** < 0.001**	124 (70.9%)	11 (68.8%)	0.86
Major VTE				** < 0.001**			0.42
Proximal DVT	47 (24.0%)	23 (23.0%)	24 (25.0%)		38 (21.7%)	6 (37.5%)	
PE only	48 (24.5%)	21 (21.0%)	27 (28.1%)		46 (26.3%)	2 (12.5%)	
PE with DVT	44 (22.4%)	13 (13.0%)	31 (32.3%)		40 (22.9%)	3 (18.8%)	
Isolated distal DVT	57 (29.1%)	43 (43.0%)	14 (14.6%)		51 (29.1%)	5 (31.2%)	
Unprovoked VTE	113 (57.7%)	49 (49.0%)	64 (66.7%)	**0.012**	104 (59.4%)	8 (50.0%)	0.46
Malignancy	15 (7.7%)	4 (4.0%)	11 (11.5%)	**0.050**	12 (6.9%)	3 (18.8%)	0.091
Smoker	31 (15.9%)	17 (17.2%)	14 (14.6%)	0.62	27 (15.5%)	3 (18.8%)	0.73
Previous VTE	40 (20.4%)	6 (6.0%)	34 (35.4%)	** < 0.001**	37 (21.1%)	1 (6.2%)	0.15
Family history VTE (1^st^ degree)	35 (17.9%)	18 (18.0%)	17 (17.7%)	0.96	29 (16.6%)	3 (18.8%)	0.82
Inherited thrombophilia^†^	36 (18.4%)	14 (14.0%)	22 (22.9%)	0.11	31 (17.7%)	4 (25.0%)	0.47
Antiphospholipid syndrome^‡^	3 (1.5%)	0 (0.0%)	3 (3.1%)	0.075	3 (1.7%)	0 (0.0%)	0.60
Days from VTE diagnosis to blood collection, median (IQR)	79.0 (41.0, 114.0)	74.5 (38.0, 104.0)	89.5 (47.0, 123.0)	0.12	79.0 (44.0, 115.0)	90.5 (41.0, 141.0)	0.58
Fibrinogen (g/L), median (IQR)	3.6 (3.0, 4.4)	3.6 (3.0, 4.2)	3.7 (3.0, 4.6)	0.46	3.5 (2.9, 4.4)	3.9 (3.6, 4.4)	0.14
D-dimer (ng/mL FEU), median (IQR)	0.3 (0.3, 0.4)	0.3 (0.3, 0.4)	0.3 (0.3, 0.5)	**0.008**	0.3 (0.3, 0.4)	0.3 (0.2, 0.7)	0.77
OCP (units), median (IQR)	39.6 (32.8, 47.8)	39.9 (33.5, 46.1)	39.4 (30.7, 50.3)	0.76	38.4 (31.9, 47.1)	42.5 (38.2, 54.0)	0.075
OHP (units), median (IQR)	9.3 (6.9, 13.3)	9.9 (7.2, 13.2)	9.0 (6.9, 13.3)	0.47	9.1 (6.8, 13.1)	10.5 (8.2, 15.3)	0.21
OFP (%), median (IQR)	75.6 (70.9, 79.2)	75.6 (71.1, 79.2)	75.6 (69.4, 79.9)	0.94	75.7 (70.8, 79.5)	76.1 (72.3, 79.0)	0.93

*SD* standard deviation; *IQR* interquartile range; *VTE* venous thromboembolism; *DVT* deep vein thrombosis; *PE* pulmonary embolism; *FEU* fibrinogen equivalent units; *OCP* overall coagulation potential; *OHP* overall haemostatic potential; *OFP* overall fibrinolytic potential

P-values in bold signify p < 0.05

^*^Anticoagulation discontinued vs indefinite anticoagulation

^#^Recurred VTE vs without VTE recurrence

^^^Five patients developed recurrent VTE prior to blood sampling – these patients were excluded from the recurrence analysis

^†^Inherited thrombophilia denotes the presence of any of the following: factor V Leiden mutation, prothrombin gene mutation, protein C deficiency, protein S deficiency or antithrombin deficiency

^‡^Antiphospholipid syndrome was diagnosed according to the Sydney criteria [21]

### VTE patients vs healthy controls

Table 2 displays the OHP assay results for the 196 VTE study patients, compared with 144 previously recruited healthy subjects. After multivariate analysis adjusting for age and sex, OCP and OHP parameters remained significantly higher in VTE patients, and OFP% significantly lower, compared to healthy subjects.

**Table 2:** Baseline characteristics and OHP results for healthy controls and VTE patients

	Healthy controls	VTE patients	p-value^*^
N	144	196	
Age, median (IQR)	42.0 (24.5, 57.0)	57.0 (47.0, 67.0)	** < 0.001**^**#**^
Male (%)	50 (34.7%)	103 (52.6%)	** < 0.001**^**#**^
Fibrinogen (g/L), median (IQR)	2.9 (2.5, 3.5)	3.6 (3.0, 4.4)	** < 0.001**
D-dimer (ng/mL FEU), median (IQR)	0.2 (0.2, 0.3)	0.3 (0.3, 0.4)	** < 0.001**
OCP (units), median (IQR)	34.5 (29.0, 43.3)	39.6 (32.8, 47.8)	** < 0.001**
OHP (units), median (IQR)	6.4 (4.8, 9.5)	9.3 (6.9, 13.3)	** < 0.001**
OFP (%), median (IQR)	81.1 (77.5, 84.1)	75.6 (70.9, 79.2)	** < 0.001**

*SD* standard deviation; *IQR* interquartile range; *FEU* fibrinogen equivalent units; *OCP* overall coagulation potential; *OHP* overall haemostatic potential; *OFP* overall fibrinolytic potential

P-values in bold signify p < 0.05

^*^Adjusted for age & sex

^#^Univariate

### VTE subtypes

In the overall study population (n = 196), the mean OCP, OHP and OFP parameters were not significantly different in patients following provoked versus unprovoked VTE (OCP 40.08 vs 40.83 units, p = 0.65; OHP 10.8 vs 10.55, p = 0.77 units; OFP 74.06 vs 74.66%, p = 0.62), or major versus minor VTE (OCP 40.87 vs 39.70 units, p = 0.52; OHP 10.92 vs 10.00 units, p = 0.30; OFP 74.14 vs 75.08%, p = 0.46).

Within the subgroup of 100 patients who subsequently discontinued anticoagulation, patients with unprovoked index VTE (n = 49) displayed significantly higher OCP than provoked VTE (43.06 v 38.70 units, p = 0.034) but no differences for OHP (11.35 v 10.01 units, p = 0.15) or OFP (74.01 v 74.88%, p = 0.55). Patients with major VTE index event (n = 57) also displayed higher OCP compared to minor VTE (42.72 v 38.34, p = 0.035) but no significant differences for OHP (11.34 v 9.79, p = 0.10), or OFP (74.03 v 75.00, p = 0.50).

### VTE recurrence

At the time of this interim analysis, there was a combined total follow-up time of 443.20 patient years, during which there were 16 VTE recurrences, of which 11 were unprovoked events and 10 were major VTE recurrences. Four recurrent VTE occurred during anticoagulation. Baseline characteristics and the OHP assay results for 191 VTE patients with and without VTE recurrence are displayed in Table 1. There were no significant differences in the OHP assay results of those with had recurrent VTE compared with those who did not recur.

### Time to event analysis

**Fig. 3:**
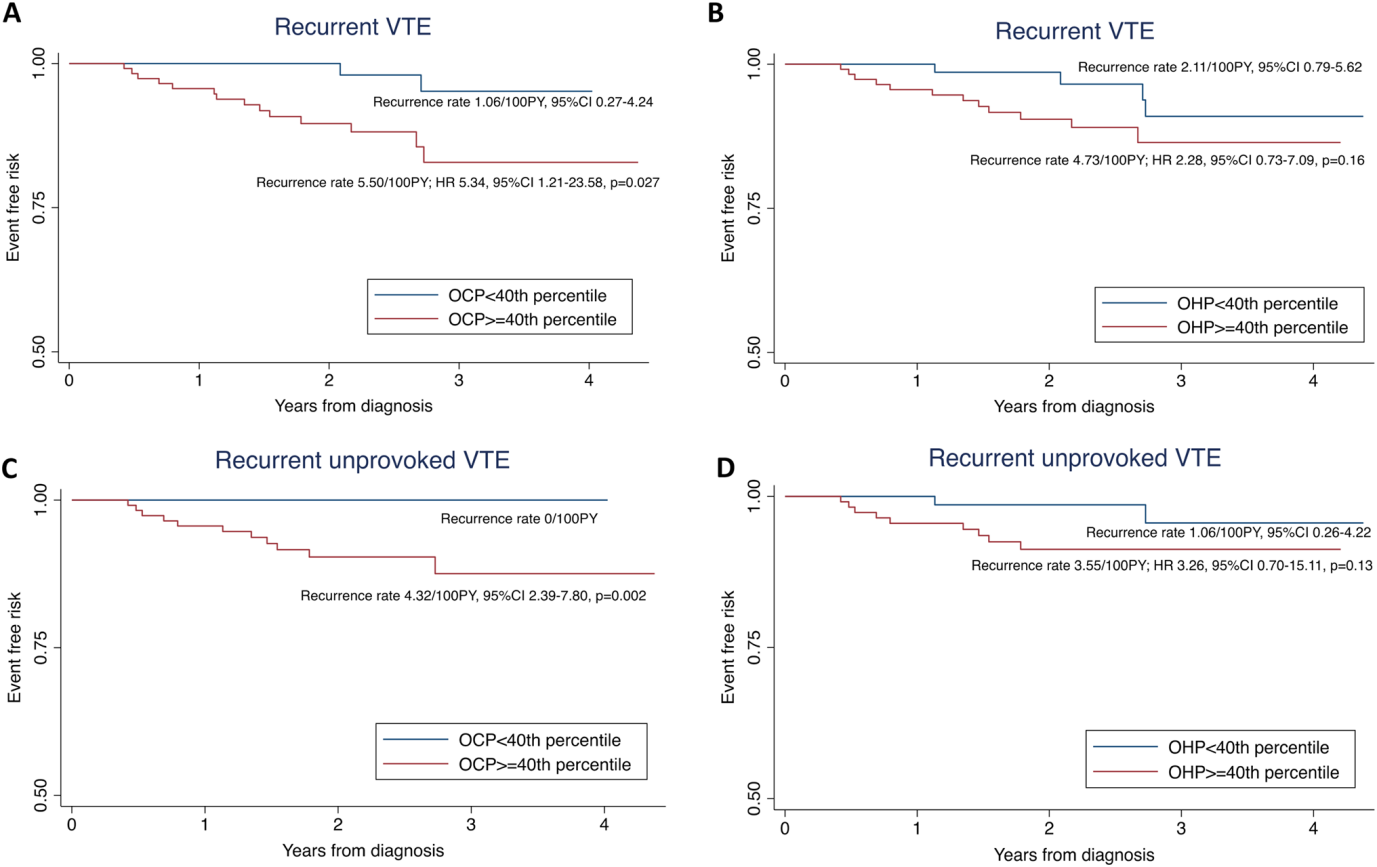
**A**–**D** Kaplan–Meier curves of recurrent VTE according to OCP and OHP in all patients (n = 196). **A**, **B** Time to event analysis for patients with all VTE recurrences (n = 16); **C**, **D** Time to event analysis for patients with unprovoked VTE recurrences (n = 11)

Time-to-event analysis of the entire cohort of 191 patients showed that an OCP cut-off corresponding to the 40^th^ percentile of OCP results was able to stratify recurrent VTE events (n = 16) (Hazard ratio (HR) 5.34, 95% confidence interval (CI) 1.21–23.58, p = 0.027) (Fig. 3A). All recurrent unprovoked VTE (n = 11) occurred above the OCP 40^th^ percentile (recurrence rate 4.32 per 100PY, 95% CI 2.39–7.80, p = 0.002) (Fig. 3C). OHP at the 40^th^ percentile, on the other hand, was not able to stratify for recurrent events with statistical significance. (Fig. 3B, D).

**Fig. 4:**
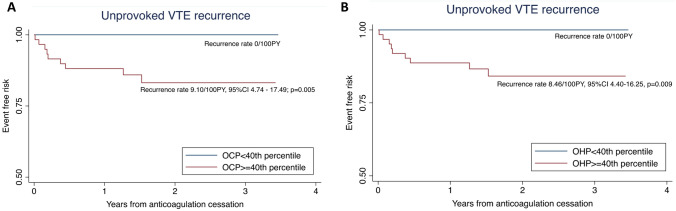
**A**, **B** Kaplan–Meier curves of unprovoked recurrent VTE (n = 9) in patients without long-term anticoagulation prophylaxis (n = 97). **A** OCP threshold at 40th percentile; **B** OHP threshold at 40th percentile

In the time-to-event analysis of the subgroup of patients who subsequently discontinued anticoagulation (n = 97) (Fig. 4), all 9 unprovoked VTE recurrences occurred above the OCP 40^th^ percentile cut-off, corresponding to a recurrence rate of 9.10 per 100PY (95% CI 4.74–17.49, p = 0.005) (Fig. 4A). The OHP 40^th^ percentile was also a significant cut-off, above which all unprovoked VTE recurrences occurred, corresponding to a recurrence rate of 8.46 per 100PY (95% CI 4.40–16.25, p = 0.009) (Fig. 4B). No deaths occurred in this subgroup of patients.

**Fig. 5:**
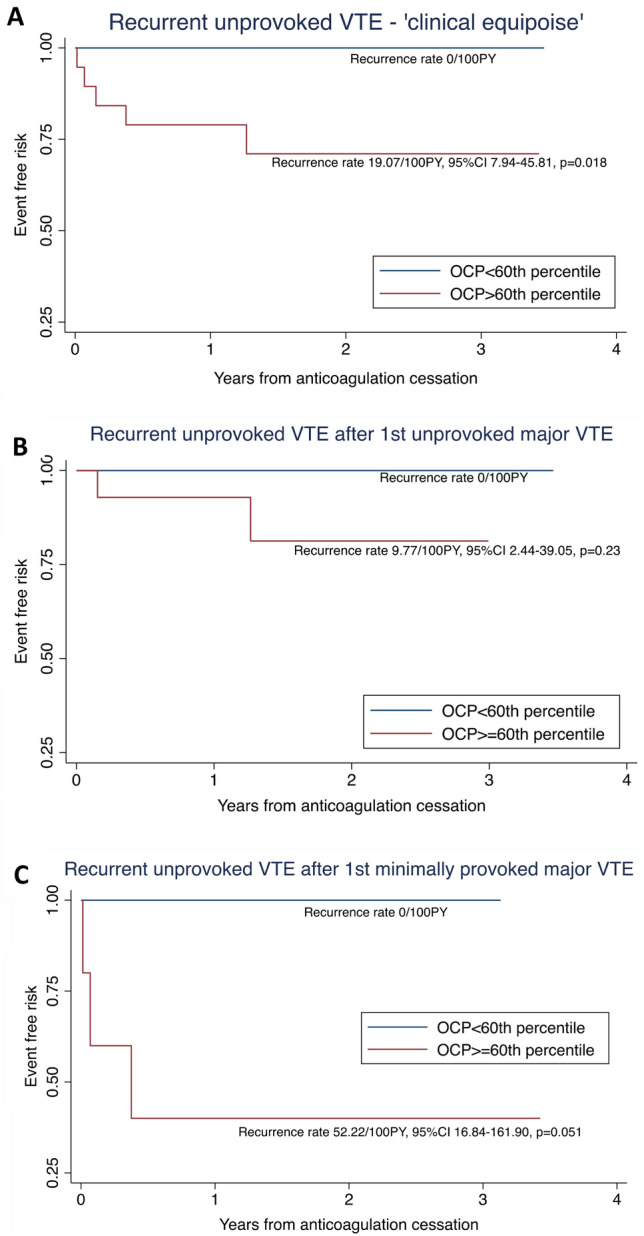
**A**–**C** Kaplan–Meier curves of unprovoked VTE recurrence according to OCP in patients without long-term anticoagulation, and in ‘clinical equipoise’. **A** ‘clinical equipoise’ (n = 34, recurrences = 5); **B** after first unprovoked major VTE (n = 25, recurrences = 2); **C** after 1st minimally provoked major VTE (n = 9, recurrences = 3)

Figure 5 displays the time-to-event analysis of patients who discontinued anticoagulation and were in ‘clinical equipoise’ with regards to VTE recurrence risk. This group of 34 patients comprised 25 patients following the first unprovoked major VTE, and 9 patients following a first minimally provoked major VTE. Within this group, there were 5 unprovoked recurrent VTE events, all of which occurred in patients with OCP above the 60^th^ percentile and corresponding to a recurrence rate of 19.07 per 100 patient-years (95% CI 7.94–45.81, p = 0.018) (Fig. 5A). The OHP parameter was not able to risk stratify for unprovoked VTE recurrence with statistical significance.

There were 62 patients not classified in the ‘clinical equipoise’ group, of which 3 unprovoked VTE occurred following IDDVT (n = 41), and 1 unprovoked VTE following provoked major VTE (n = 21). All 4 unprovoked recurrences occurred above a OCP cut-off of 36.25, equivalent to the 40^th^ percentile and corresponding to a recurrence rate of 6.73 per 100 patient-years (95% CI 2.53–17.94, p = 0.058).

**Fig. 6:**
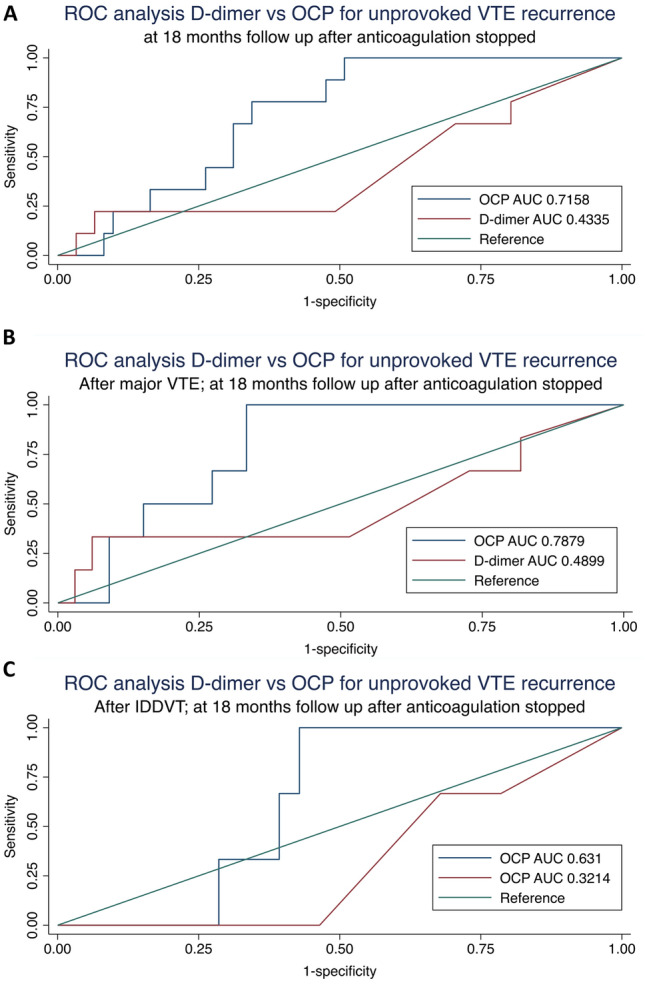
**A**–**C** ROC curve analysis for D-dimer and OCP in patients at 18 months from anticoagulation cessation with outcome of unprovoked VTE recurrences. **A** all (n = 70); **B** after major VTE (n = 39); **C** after IDDVT (n = 31)

Figure 6 displays ROC analysis performed for D-dimer and OCP to examine their ability to predict unprovoked recurrent VTE in patients with at least 18 months follow-up from anticoagulation cessation (n = 70), and further subdivided to those following major VTE (n = 39) and IDDVT (n = 31). D-dimer performed poorly across all patient subgroups with low area under the ROC curve (AUC) (Figs. 6A–C). OCP demonstrated the best performance in the subgroup of patients following major VTE with an AUC of 0.79 (Fig. 6B).

## Discussion

To the best our knowledge, this pilot study is the largest to study fibrin generation and fibrinolysis using the OHP assay in VTE patients, and the first to evaluate this assay during the acute anticoagulation period, particularly in its role to risk stratify for VTE recurrence. We demonstrate that despite being anticoagulated, patients with VTE possess higher fibrin generation capacity and reduced fibrinolytic potential compared to healthy subjects. The ability to interrogate coagulation parameters whilst anticoagulated is of practical importance and allows risk assessment without the need to cease anticoagulation, particularly in patients at high risk of VTE recurrence. This is advantageous over other global coagulation assays such as thromboelastography and thrombin generation assays, which are significantly impacted by the effect of anticoagulation.

Our findings demonstrating hypercoagulability in VTE patients are consistent with previous studies using the OHP assay in hypercoagulable disease states such as VTE, cardiovascular disease [18], pregnancy [23], vasculitis [24] and antiphospholipid syndrome [19]. Chow et al. [22] studied OHP in 67 long-term PE survivors who were no longer receiving therapeutic anticoagulation (mean sample collection 7.9 years after PE). Compared to 20 healthy controls, the mean OCP and OHP were significantly elevated, and OFP significantly lower in PE patients. Antovic et al. [16] demonstrated higher OHP and prolonged clot lysis time in 88 women with previous pregnancy-associated VTE compared with 25 healthy young women. Our study is unique, however, as we performed the assay on anticoagulated individuals.

Using time-to-event analysis, we found the 40^th^ percentile of OCP in the entire cohort (Fig. 3) to be discriminatory for patients at higher risk of all VTE recurrences (HR 5.34, 95% CI 1.21–23.58, p = 0.027) as well as unprovoked VTE recurrence (recurrence rate 4.32/100PY, 95% CI 2.39–7.80, p = 0.002). In patients who subsequently discontinued anticoagulation, the 40^th^ percentile for both OCP and OHP was able to stratify the risk of developing unprovoked VTE recurrence (Fig. 4). Within the ‘clinical equipoise’ subgroup in this study (n = 34), where the role of long-term anticoagulation requires further refinement particularly at an individual level, all 5 unprovoked VTE recurrences occurred above the 60^th^ OCP percentile. Using this cut-off, 16 (47.1%) of the ‘clinical equipoise’ subgroup could be deemed low risk for unprovoked VTE recurrence and may potentially not need long-term prophylaxis. In addition, OCP was found to be superior to D-dimer at predicting unprovoked VTE recurrence, particularly in patients following an index major VTE event (ROC AUC of 0.79 vs 0.49, p = 0.037). These pilot results would suggest that the OHP assay, despite the patients being anticoagulated, may be discriminatory for unprovoked VTE recurrence risk, particularly for those in ‘clinical equipoise’.

The evidence supporting reduced fibrinolysis as a predictor of VTE recurrence has been conflicting. Other studies of plasminogen activator inhibitor-1 (PAI-1) [25, 26] and clot lysis time (CLT) [12, 26] have been mixed and have not translated to routine clinical use. The differences may be attributed to varying study designs and inclusion criteria Further study within this area is warranted. The OHP assay has the advantage of being able to simultaneously interrogate the fibrin generation and fibrinolytic pathways, with the OHP parameter being a representative of the balance between fibrin generation and lytic capacity within one individual. In this study, the OHP 40^th^ percentile was the threshold above which all unprovoked VTE recurrences occurred within the subgroup of patients who subsequently discontinued anticoagulation.

There are several limitations to our study. Given the pilot nature of this study, the study population was relatively small. This was further compounded by a high proportion of patients (49%) who went on to indefinite anticoagulation, which may have limited the number of VTE recurrences. The high proportion of long-term anticoagulation may reflect current clinical practice following evidence supporting the use of long-term low-dose direct oral anticoagulants [27, 28]. Despite small numbers, our pilot results have been able to demonstrate that the OHP assay, particularly the OCP parameter, is able to risk stratify VTE recurrence risk and warrant validation in larger studies. The VTE subjects were significantly older (57 vs 42 years, p < 0.001) and more likely male (52.6% vs 34.7%, p < 0.001) compared to the healthy subject group. However, differences in the OHP assay results remained significant after adjusting for age and sex by multivariate analysis and these appear to discriminate for the risk of VTE recurrence despite testing occurring whilst on anticoagulation (see Table 2). Our OHP assay was modified to have a lower dose of thrombin (0.006U/mL vs 0.04U/mL) compared to the original assay developed by He et al. [14] and hence may not be directly comparable with other studies [19]. Our rationale for using the lower thrombin concentration was to enhance the sensitivity of the OHP assay to detect increased fibrin generation capacity in patients with prothrombotic tendency. The tPA concentration used was unchanged however, and in excess of physiological concentrations. This may have contributed the OHP and OFP parameters being unable to detect differences in VTE recurrence risk within the entire patient cohort and the ‘clinic equipoise’ subgroup.

In conclusion, we have demonstrated in this pilot study the ability of the OHP assay to detect a hypercoagulable and hypofibrinolytic state in anticoagulated patients following VTE, compared to healthy subjects. In this interim analysis of an ongoing study, the OHP assay was also able to discriminate the risk of VTE recurrence and suggests this assay may hold promise as an adjunct to further refine risk assessment allowing for more individualised targeting of long-term anticoagulation prophylaxis. Further long-term and larger studies evaluating fibrin generation assays are required.
